# Compassionate use of orphan drugs

**DOI:** 10.1186/s13023-015-0306-x

**Published:** 2015-08-21

**Authors:** Hanna I. Hyry, Jeremy Manuel, Timothy M. Cox, Jonathan C. P. Roos

**Affiliations:** Department of Medicine, University of Cambridge, Cambridge, CB2 0QQ UK; European Gaucher Alliance, UK Gaucher’s Association, 340 West End Lane, London, NW6 1LN UK

**Keywords:** Orphan drugs, Compassionate use, EU, Legal, Ethical and pragmatic arguments

## Abstract

**Background:**

EU regulation 726/2004 authorises manufacturers to provide drugs to patients on a temporary basis when marketing authorisation sought centrally for the entire EU is still pending. Individual Member States retain the right to approve and implement such ‘compassionate use’ programmes which companies will usually provide for free. Nevertheless some companies have opted not to partake in such programmes, in effect restricting access to drugs for patients in need. Here we survey the state of compassionate use programmes in the EU with particular reference to the rare disease field, and provide legal and ethical arguments to encourage their increased compassionate use in the EU and beyond. We contend that if enacted, these recommendations will be mutually beneficial to companies as well as patients.

**Methods:**

Requests for information from the European Medicines Agency were made under the UK Freedom of Information Act 2000. Legal, ethical and economic/pragmatic analysis identified means by which provision of therapy in compassionate use programmes might be increased.

**Results:**

More than 50 notifications of compassionate use programmes have been submitted to the EMA by Member States since 2006. About 40 % relate to orphan drugs. As there is a compulsory register of programmes but not of outcomes, their success is difficult to evaluate but, for example, the French programme expedited treatment for more than 20,000 (orphan and non-orphan) patients over a period of three years.

**Conclusion:**

Compelling self-interested, legal and ethical arguments can be mounted to encourage manufacturers to offer therapies on a compassionate use basis and these are often equally applicable to provision on a humanitarian aid basis. The EU’s compassionate use programmes are instrumental in ensuring continuity of access to drugs until approval and reimbursement decisions are finalised. We propose the creation of a registry of drugs offered on a compassionate use basis; further transparency would allow such programmes to be evaluated and direct patients to sources of treatment.

**Electronic supplementary material:**

The online version of this article (doi:10.1186/s13023-015-0306-x) contains supplementary material, which is available to authorized users.

## Background

‘Compassionate use’ refers to a manufacturer providing a therapeutic product, often without charge, to patients in need on a temporary basis. When phase 3 drug trials are coming to completion or when marketing authorisation is pending, this practice of making a drug available on compassionate grounds is quite frequent in the United States and European countries. A noteworthy example was provided by Glaxo-Wellcome in the 1990s: while phase 3 clinical trials were in progress, the company donated its AIDS drug, ziduvidine, free of charge to 22,000 patients [1]. In the setting of rare diseases, the Genzyme Corporation generously donates imiglucerase for life-saving treatment of hundreds of severely affected patients with Gaucher Disease in three large-scale international compassionate use programmes.

Not all companies contribute (substantially) to compassionate initiatives of this kind, thus raising the question as to why *should* a profit-making enterprise donate a drug? This article surveys the state of compassionate use programmes in the EU, and explores how provision of commercial drugs compassionately by a pharmaceutical company can be viewed as an activity of enlightened self-interest; we also set out legal and ethical arguments which, we argue, compel such provision. We draw here on examples principally from the European Union and medicines for rare, ‘orphan’ disorders as this is our area of expertise; nonetheless the arguments adduced apply equally to treatments for more common debilitating or life-threatening conditions.

In the EU and beyond, compassionate use can also offer a critical life-line to patients where a country struggles to offer basic medical care and is unable to purchase expensive life-saving drugs. There may also be extraordinary circumstances such as earthquakes which disrupt medical services and transport routes, or shortages of supply due to production difficulties.

The detailed definitions of and differences between i) compassionate use (product *not* yet licensed, or newly licenced), ii) off-label use (product licensed but prescribed for a different indication) and iii) clinical trials (legal requirement prior to gaining marketing authorisation) are further summarised in Additional file 1: Table S1. Humanitarian provision is often distinguished from compassionate use as explained further in Additional file 1: Table S1. However, we also test the soundness of this distinction and contend that the arguments expounded below apply in many instances to humanitarian aid. For the purposes of this article they are thus treated similarly.

Of note, the EU is piloting within the compassionate use framework an ‘adaptive pathways approach’. This involves an iterative and dynamic approach to collection of evidence and consequent licence adaptations that will facilitate speedy access to life-saving medications – drugs which may, for example, involve conditional regulatory approval [2]. If the approach allows life-saving medications to gain marketing authorisation more quickly, this may ultimately shorten the duration for which provision based on compassionate use is required.

## Methods 

This article contains a legal, ethical and economic/pragmatic analysis to identify means by which provision of therapy in compassionate use programmes might be increased, drawing on examples principally from the European Union and medicines for rare, 'orphan' disorders.  The article also presents and analyses data obtained from the European Medicines Agency under the UK Freedom of Information Act 2000 concerning compassionte use programmes.

### The state of ‘compassionate use’ in the EU

To minimise health risk to patients, medicines must obtain marketing authorisation before being sold in the EU (Directive 2001/83). After completion of trials demonstrating safety, tolerability and efficacy of a new agent, final authorisation can still take many months to achieve; in the French experience 11 months on average [3]. There follows a further time lag for distribution of the drug into the EU market. This period can range from a few months in Germany and the UK (which do not regulate launch prices) to 11 and 12 months in France and Italy, where reimbursement pricing has to be negotiated first. [3] Indeed, individual Member States retain autonomy and may delay or not approve a drug for local reimbursement. Once an authorised and approved drug is available, patients may struggle to secure insurance cover or the drug may not yet be approved by hospital formularies, adding further delay.

We contend elsewhere that public and private health providers should offer life-saving treatments once they are thus available in the market [4, 5]. However, if a patient has no alternative treatment available in the meantime, they will be deprived effective treatment of their disease. This can be a particular difficulty for patients suffering from rare diseases for two reasons. First, the historic neglect of rare diseases means few if any treatments exist. Second, once an orphan drug gains marketing authorisation, it secures a statutory monopoly position which precludes the authorisation of other treatments for 10 years, subject to limited exceptions such as a subsequent treatment being safer, more effective or otherwise clinically superior [6]. The patient is therefore reliant on that treatment being made available.

In response, EU Regulation 726/2004 creates an explicit exemption from the marketing authorisation requirement for compassionate use (see Additional file 2: Table S2 for details including the official definition of compassionate use pursuant to Article 83 [7-10]). The aim of the exemption is thought to be temporary until the treatment is launched in the market, although we note that Article 83 makes no express reference to the temporary basis (see Additional file 2: Table S2). A model for the Regulation came *inter alia* from France in the early 1990s in response to the AIDS pandemic while clinical trials were still ongoing [1]. The French exemption programme shortened the time that patients wait to start treatment, by 36 months [3]; since 2007 this exemption programme has expedited the treatment of more than 20,000 patients treated with over 200 drugs [11]. But the stance taken by EU Member States to compassionate use varies greatly, with for example Ireland, Sweden and the UK having no formal compassionate use programme and Hungary having no programme at all [12].

**Fig. 1:**
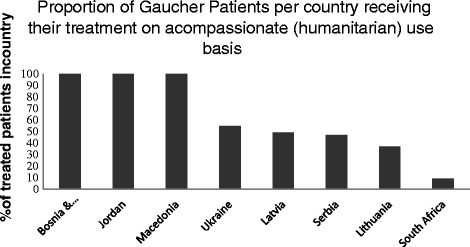
Proportion of Gaucher Patients per country receiving their treatment on a compassionate (humanitarian) use basis. Data from selected countries showing the proportion of Gaucher Patients receiving treatment on a compassionate (humanitarian) use basis

Since the enactment of the EU Regulation, which allows but does not require countries to have such compassionate use programmes, Member States have sent altogether 41 notifications of compassionate use programmes, 17 (41 %) of which are for orphan drugs (Additional file 3: Table S3 [13]). Figure 1 illustrates, with Gaucher Disease as an example, that some EU and other countries rely greatly on compassionate use for treating their patients [14].

**Fig. 2:**
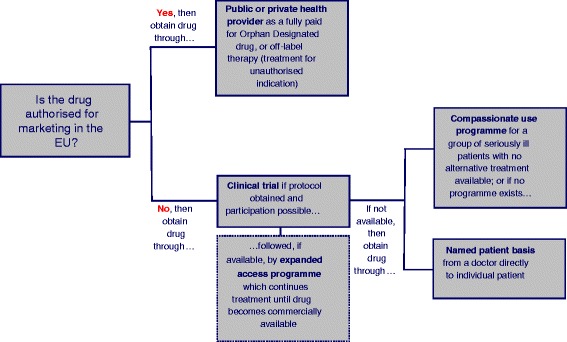
Pathways for accessing an orphan therapy in the EU. A schematic illustration of how an orphan therapy can be accessed in the EU

The pathways for accessing an orphan therapy in the EU are summarised in Fig. 2.

### Justification for compassionate provision

We suggest that it can be in the interest of pharmaceutical companies to offer a drug to patients on a compassionate use basis. We contend that taken together, the pragmatic and ethical arguments explored below present a persuasive case for compassionate use provision.

First, such provision tends to fit squarely within corporate social responsibility programmes.

Second, while the primary aim of compassionate use provision is not data generation, the experience can provide invaluable clinical data particularly in rare diseases which are investigated in trials which often comprise relatively few patients. Clinical data obtained from compassionate use supply further ‘real world’ information about responses in patients who may not meet rigid entry criteria for clinical trials. Clearly, late-phase clinical trials in this field are designed principally to investigate efficacy using protocols that regulators approve but for this reason, often exclude patients with advanced disease or co-morbidities. All EU countries maintain a register of unwanted effects arising from compassionate use programmes. These may allow identification of unwanted effects and interactions with other prescribed drugs at an early stage, thereby refining the indications and guidance for prescribing as well as the costs of litigation [15, 16].

Of note, the new EU Clinical Trials Regulation, signed into law in April 2014 and expected to come into force in 2016, creates greater transparency and requires drug companies to publish detailed trial data. Companies might therefore opt to run smaller trials on those highly selected patients most likely to show benefit (provided they can continue to demonstrate that, despite the smaller size, the data are robust), and then provide their drug on a compassionate use basis for the remainder of patients whilst being reimbursed (in most states) and not requiring declaration of those results as they would nor form part of a trial. This development should be monitored, as the Regulation states that compassionate use programmes are not a substitute for clinical trials [17] (see also Additional file 2: Table S2).

Third, compassionate use may provide rapid access to revenue where national legislation allows for reimbursement. In France, hospital pharmacies can pay the full price to manufacturers for compassionate use treatments – indeed companies set a 12 % premium on therapies sold through this programme [3]. On the other hand, Germany amended its legislation for compassionate use in 2009 so that such agents must be provided free of charge [11]. The authors appreciate that the example of policy action in France is an instance of a government “taking charge” of a compassionate use programme to allow for reimbursement. Companies and patients may wish to lobby for similar legislation in other countries to yield a programme which visibly benefits both companies and patients.

Fourth, for commercial reasons a company may prefer to donate a drug rather than sell at a discounted rate to individual countries [6]. Any price discrepancy could prompt requests from more affluent countries to reduce their drug cost. With such a precedent, a company might well find it difficult if not impossible to justify price discrimination – a state of affairs in which it might eventually have to offer the discounted price to all patients in the EU, and with significant consequences for profits. Donating the drug for free avoids downward harmonisation of pricing.

Fifth, there are often long-term strategic advantages for companies and their political counterparts involved in provision of health care in countries which are presently too poor to pay for expensive orphan medicinal products. Administration of biologic agents and management of chronic diseases requires investment in expertise and medical services beyond basic pharmacy. Development of the required infrastructure and staff facilities is predicated on bilateral investments contracted with the investing company – and an accompanying platform for enhanced and realistic market investment, thereby helping to foster a market where previously there was none. This has been the case for Gaucher disease in Romania where a humanitarian aid programme has now created a viable market in which companies are now generating revenue.

Sixth, offering a drug on a compassionate basis can help establish an early market presence in affluent countries as it allows doctors to familiarise themselves with the new drug and manufacturers to establish relationships with specialist doctors, government departments, hospitals, charities, and where relevant, insurance companies, as part of market development and, ultimately, commercial dominance. This can be the case particularly during an “expanded access programme” meaning a period after the trial ends and before the drug becomes available for distribution. Similarly, the Regulation ensures that patients taking part in a compassionate use programme have access to the treatment during the period between authorisation and placing on the market (see Additional file 2: Table S2).

Some companies cite difficulties in entering a country with the given agent as a reason for not engaging with compassionate use provision; but patient organisations may have on-the-ground presence and be able to make introductions – and otherwise facilitate entry through activities in popular media and occasionally through political lobbying.

Seventh, and related, compassionate use establishes good will with the stakeholders listed above, allowing networks to protect the manufacturer should a competitor seek to sell an alternative therapy for the same condition: patients and other involved parties would probably wish to buy the drug from the company and individuals already operating in the market, unless a new agent is demonstrably superior. This argument does not envisage sinister “blocking” of the market, but simply reflects on the practical reality that a patient and his physician are likely to prefer an existing therapy that is effective to a new, untried therapy which may be of uncertain efficacy and unknown side effects.

On this last point, subsequent market entrants may also benefit from contributing to compassionate programmes. As an example, when global production of imiglucerase was compromised in 2009, two rival manufacturers with licensed enzyme preparations that had yet to receive marketing approval were asked by EMA (and the FDA) to make up the shortfall, requiring them to accelerate their development, regulatory approval and scale-up of their bioreactor production facilities [18]. In effect, this meant investing in production and distribution of a life-saving treatment in the absence of marketing authorisation (thus meeting the definition of compassionate use provision in Article 83(2) – see Additional file 2: Table S2 and [8]). This was initially a commercially attractive opportunity for these manufacturers; they later secured marketing authorisation for their drug as part of the negotiations with the authorities so that they could begin to sell their drug in the EU and US.

It is noteworthy, however, that while taliglucerase-alfa, the enzyme preparation for Gaucher disease developed through a novel protein expression system in plant cells by Protalix Biotherapeutics gained approval by the FDA, it was not approved in Europe by EMA. Here, the precedent and hence gain of marketing exclusivity as an orphan medicinal product was lost to velaglucerase-alfa, the biologic now marketed by Shire. Thus in the unusual setting of late-phase competition in an ultra-orphan disease, the incentive proved to be far less clear for companies who do not succeed in securing authorisation, as in this case where there was no prospect of attaining any share of the market after the compassionate use provision ended.

These examples of what one might call self-interested reasons for compassionate use do not appear to have convinced all manufacturers to initiate (or contribute to) compassionate use programmes. Here we explore several legal and ethical principles that can be adduced to justify more extensive introduction of the practice.

### Legal mechanisms to encourage compassionate use

We contend elsewhere that human rights and disability legislation require governments to reimburse the costs of orphan medicinal treatments [4], but these appeals ring hollow if a country is too poor to offer its citizens more than the most rudimentary care. At first, it also seems difficult to argue that a company has a legal obligation to give away drugs for free. However, a case can be made for how such an obligation can be created on three levels: (i) regional or government level, (ii) between two or more orphan drug manufacturers, and (iii) between the manufacturer and patients. These are set out in detail in Additional file 4: Table S4 and [19]. In brief:

i) **Mechanisms on a regional and governmental level**. The EU could amend its orphan drug legislation to state that orphan designation after marketing authorisation is only maintained if the company undertakes to ensure provision of drug (free or at a reduced rate) for those EU Members States with a Gross Domestic Product below a certain low limit (for example Romania and Bulgaria or the countries in Fig. 1), or those States experiencing severe recession (for example Greece where treatment cessation is feared to have led to deaths [20]) or are otherwise demonstrably unable to pay for the treatment.

As this could place a considerable burden on a company, the legislation could permit tax relief for costs associated with compassionate use; small and medium companies already obtain fee reductions on administrative fees under Regulation 2049/2005. We would however expect resistance to such amendment: the perception is that manufacturers prefer not to disclose the details of their compassionate provision for fear of being asked to provide the drugs for free more widely, and are therefore unlikely to want to accept a legal obligation which makes such programmes official and mandatory.

ii) **Mechanisms on a manufacturer level.** The terms of agreement of industry groups such as the European Federation of Pharmaceutical Industries and Associations (EFPIA) and similar organisations around the world could specify that, where more than one company sells a drug for the same condition, each manufacturer must make its best effort to partake in a compassionate use programme. The details of provision could be governed by a non-disclosure agreement between the manufacturers if they are concerned that publicity around the details of provision encourages patient organisations to demand free provision more widely. Agreements to share the burden of provision will however not assist patients where only a single drug exists for a condition, which is the case, almost by definition, for most orphan diseases.
iii) **Mechanisms on a patient level**. Action by patient organizations is best. EU Regulation 726/2004 provides that patients taking part in a compassionate use programme continue to have access to the treatment between the time when the drug gains marketing authorisation and its full entry on the market (Art 83(8)). Patients who participate in *clinical trials* (distinct from compassionate use – see Fig. 2) sometimes partake in an expanded access programme when the trial ends and before the drug becomes available for distribution, but there is no legal obligation on the company to offer such continued expanded access programme. Individual patients wishing to join a clinical trial have little power to negotiate the terms under which they participate.

However, there are influential umbrella organizations often representing large populations of patients: EURORDIS in the EU or NORD in the US are examples. These organisations have strong bargaining positions, as do charities underwriting the costs of research trials. Moreover, the EU Clinical Trials Directive 2001/20 requires that each trial participant gives his or her consent to the conditions and risks of the trial in writing. Patient organisations could demand that all consent agreements entered into going forward contain a new clause that is identical between individual consent agreements. The clause could specify that each trial participant or disease registry member, including those on a placebo, are to be given the drug (or, in the case of multi-arm trials, the most effective drug) for free following a trial until the patient’s public or private health plan covers it. Such a clause would be consistent with the Declaration of Helsinki of the World Medical Association setting out ethical principles regarding trials, and provides that

*In advance of a clinical trial, sponsors, researchers and host country governments should make provisions for post-trial access for all participants who still need an intervention identified as beneficial in the trial. This information must also be disclosed to participants during the informed consent process* [21]. (Emphasis added.)

Such a contract with patients or their organizations would also create an inducement for companies to negotiate rapidly a reasonable reimbursement scheme with care providers, lest they continue supplying drug for free longer than necessary. Simultaneously, safeguards would need to exist to stop companies from being taken advantage of by a care provider’s refusal or delay in paying for treatment. A company might for example be able to bring a judicial review request against a public authority’s refusal to purchase a treatment, although victory in judicial review is very uncertain and the merit of each case would have to be assessed carefully in each case [4].

Once patients have been recruited to and given consent for a given clinical trial, this bargaining chip for patient organizations and related charities disappears because the patients have already consented to take part. Early and concerted action by patient organisations is therefore essential.

### Ethical reasons for offering orphan drugs for free

The term “compassionate” use conveys the idea that companies exceptionally donate a life-saving drug because the plight of the patient (often a child) elicits a feeling of compassion. Irrespective of the reality that only a few drugs are life-saving and indeed that not all patients with rare conditions have truly life-threatening conditions, it is useful to consider whether this sentiment can be crystallized into a coherent ethical theory.

The so-called principles of “beneficence” and “rule of rescue” are not satisfactory expressions of this sentiment since they are arbitrary and place an undue burden on patients who may lack the resources to render themselves identifiable on a sufficient scale. A more objective and generally applicable alternative can be found in John Rawls’ *Theory of Justice,* which provides a cogent basis for the argument that individual countries (or their private health plans) should pay for expensive orphan drugs [5, 22]. Rawls (1921–2002) does not specifically address whether a for-profit drug company has a responsibility to patients, and perhaps extending to patients in other countries where it may not even operate. Rawls is solely concerned with justice for societies and countries and his theory of *international* justice is not concerned with the well-being of *individuals* or the arbitrariness of their fates [23]. However Kant and Aristotle are two other moral philosophers who offer reasons to donate a life-saving drug for free.

The writings of Immanuel Kant (1724–1804) are important in contemporary Western philosophy and he greatly influenced Rawls. Kant argues that morally right actions are those done for reasons that are intrinsically ‘right’ and which are commonly accepted human societal behaviours. We should give away a drug for free not because the compassionate act gives us a warm feeling, but because humans have independent value [24]. Kant’s rule of thumb is: act in such a way that you always treat humanity, whether yourself or others, not as a means but as an end [24]. Providing a life-saving drug for free on a temporary basis arguably respects humanity as an end. Refusing to provide would be disrespectful to human life as patients would suffer and some would probably die prematurely.

Aristotle (384–322 BC) argues, perhaps in a more accessible manner, that we should do what makes us happy [25]. But happiness for Aristotle was not a loose and relative concept of hedonistic pleasure or power. He defines happiness strictly: acting virtuously. Aristotle differed from Socrates and Plato in emphasising that merely having a virtue is not enough. One should also *act* virtuously. A central pillar of virtue, says Aristotle, is moderation. For example in giving money – or in our case life-saving drugs – we should neither be profligate nor miserly, for both are undesirable extremes. Instead, we should aim to be generous [25]. A drug company should establish a compassionate use programme because it is a generous, and virtuous, action.

The strength of Kant’s and Aristotle’s ideas is that they envisage compassionate use provision in and beyond the EU to patients where it is needed – in the case of treatments for Gaucher disease, most recently India, Pakistan, South America, Egypt and Africa. A company might object that business organizations cannot be regarded as natural persons capable of happiness, but of course those working for a company are, and it is their and their patients’ happiness that Aristotle would have wished to ensure.

A biopharmaceutical company might also respond that its shareholders are interested in profit, not virtue, and that profit may be used in numerous ways that are virtuous but determined by the inventors and employees of that organization. However, Aristotle promulgates the view that seeking instrumental goods – such as money – as an end goal leads away from a good life [25].

And here Aristotle perhaps inadvertently offers a perceptive management strategy: because people are fulfilled and perform best when they feel moral satisfaction [26], the company should have a practical interest in creating opportunities that promote virtuous activities on the part of its employees. It might offer for example a chance to participate in organizing a compassionate use programme for a country where patients currently receive no treatment for debilitating and life-threatening conditions.

Thus, even if a company is unmoved by the philosophy of moral rectitude, it makes business sense to heed the recommendations of Kant and Aristotle – though strictly speaking this is a reason borne out of what one might term enlightened self-interest, a matter that we have explored here under the first heading above.

On a related point, some pharmaceutical companies cite the lack of local medical expertise and basic healthcare infrastructure required for administering a given agent as a barrier to offering free compassionate treatment. Accordingly, companies could establish programmes to train those charged with prescribing and administering their life-saving drug.

The challenge is that for such programmes to be truly life-saving, they must be long-term. Delivering a treatment on an urgent, short-term basis may capture headlines but even if it were highly efficacious, the intervention will not yield permanently improved outcomes. More is required than the treatment itself: there should also be in place a system for managing and monitoring the provision of treatment to improve care over the patient’s lifetime. Development of local medical services is a critical factor in ensuring that treatment is used appropriately and is effective in the long-term. The company should also be ready to engage in protracted negotiations with authorities to secure licences and permission to import the drug as well as local health officials; often these negotiations must be accompanied by a prolonged dialogue with senior politicians, including those of ministerial rank. Companies are also now obliged to check that the pathway for providing a drug complies with the expansive anti-bribery regimens now operating in countries where drugs and other assistance are given freely as part of international aid programmes.

While the processes for achieving an altruistic purpose can be very demanding and even exasperating, ultimately a successful practical outcome is likely to reward the company in commercial terms, as well as in the ethical sense as discussed above. It may weigh positively in the balance that the real cost of compassionate use provision for a pharmaceutical company is the cost of production and distribution of the product, without having to include, for example, marketing and promotional costs.

This leads directly to another pragmatic reason, namely that a company will need the good will of regulators, medical professionals and patient organisations in securing participants for current and future drug trials, getting the drug on public and private formularies, having it prescribed, and in persuading doctors to sit on its advisory boards. Those working in healthcare or the civil service may have chosen such career at least in part because of their interest and pleasure in doing the ‘right thing’ through humanistic fulfilment. A company that is perceived as morally odious may struggle to obtain the necessary cooperation of doctors, patient organizations and the government in bringing a drug to the market or maintaining a lucrative therapeutic position when competition looms.

## Results

Compelling pragmatic, legal and ethical arguments to convince orphan and other drug companies to provide life-saving drugs where circumstances prevent patients from accessing treatment. More than 50 notifications of compassionate use programmes have been submitted to the EMA by Member States since 2006. About 40 % relate to orphan drugs. As there is a compulsory register of programmes but not of outcomes, their success is difficult to evaluate but, for example, the French programme expedited treatment for more than 20,000 (orphan and non-orphan) patients over a period of three years.

## Conclusions

In its foundation, the orphan drug legislation represents an expression of non-economic societal values which seek to realize the humanitarian purpose of providing equitable access to treatments for rare diseases independent of their rarity. Barriers to access based on ability to pay, are thus contrary to these moral precepts: by the same token, compelling incentives are needed to ensure that where an effective therapy is available, it can be obtained by those for whom it was developed.

Clearly, profit is key to the viability of any company and it would be naïve to advocate open-ended commitments to charity which would cripple balance sheets. However, introduction of tax exemptions and other measures could diminish the actual cost of compassionate provision by companies. Moreover, provision of life-saving drugs at reduced prices or free is likely to leave companies with several net gains: of favourable publicity, financial approval and fulfilled employees, as well as a contented market reverberating with grateful patients. Moreover, the action is not only generous; to give readily and to give generously is a demonstration of strength. Compassionate and related charitable programmes promulgated by pharmaceutical manufacturers are in effect an assertion of marketing confidence. This notional concept, redolent of courageous generosity, is, in effect, a cryptic statement of commercial power.

A strategic objective of the EU is to create a European platform for registration of rare diseases [27]. A publicly available EU-wide registry which lists all compassionate-use and expanded access programmes for orphan and other drugs, including the locations and numbers of patients treated, would further incentivise manufacturers to engage in compassionate provision, although we understand that there is resistance to the initiative. Companies are in any event obliged to maintain registries of patients who will be prescribed their drugs after marketing approval, as a responsibility contracted with regulatory authorities and part of their requirements for long-term pharmaco-vigilance. It may also be that publication of the details of treatment responses, safety and tolerability may lead to further demands for the provision of compassionate use by patients.

At the outset, it would be preferable to agree the terms of compassionate provision – treatment, dosage and duration. One aspect of ‘sustainability’ is thus met, since the agreement would not only give patients certainty, it would allow those treating them and their advocates to secure reimbursed treatment in good time to ensure that the treatment is not suddenly stopped Patient organisations may have a role in brokering standard contracts for compassionate use provision which capture these important elements.

## Additional files

Additional file 1: Table S1. Comparison of compassionate use, humanitarian aid, off-label use and clinical trials. A table setting out the differences between compassionate, humanitarian aid, off-label use and clinical trials. (DOCX 74 kb) Additional file 2: Table S2. Analysis of EU Regulation 726/2004. A table analysing the legal definition of compassionate use in the EU. (DOCX 80 kb) Additional file 3: Table S3. Compassionate use programmes notified to EMA by Member States arranged by year. A table of compassionate use programmes notified to EMA by Member States, obtained from EMA via a freedom of information request. (DOCX 175 kb) Additional file 4: Table S4. Legal mechanisms for achieving compassionate use provision. A table elaborating on legal mechanisms for achieving compassionate use provision. (DOCX 74 kb)
