# Supplementary material for: Compassionate use of orphan drugs
Source: Orphanet J Rare Dis. 2015 Aug 21;10:100. doi: 10.1186/s13023-015-0306-x (PMC4546220; doi:10.1186/s13023-015-0306-x)
Supplement: Additional file 1: Table S1. — Comparison of compassionate use, humanitarian aid, off-label use and clinical trials. A table setting out the differences between compassionate, humanitarian aid, off-label use and clinical trials. (DOCX 74 kb) [file 13023_2015_306_MOESM1_ESM.docx]

**Additional file 1: Table S1. Comparison of compassionate use,** **humanitarian aid, off-label use and clinical trials (adapted** **and developed from Whitfield et al., 2010[6])**

| **Topic** | **Compassionate use** | **Humanitarian aid** | **Off-label use** | **Clinical trials** |
| --- | --- | --- | --- | --- |
| **Summary** | Treatment for a life-threatening condition on a temporary and unlicensed (or newly licensed) basis where no better treatment yet exists (see Supplementary File 2 for the official definition enshrined in EU Law) | Treatment of a life-threatening condition with a licensed product, sometimes thought to differ from compassionate use in that there is **no** **prospect of a financial return**.  This distinction between compassionate use and humanitarian aid is open to challenge on the following basis:   - Humanitarian aid can also lead to profitable sales, as has been the case for example for Gaucher treatment in Romania. - In fact, many manufacturers maintain unofficially that they engage in humanitarian aid provision only in countries where they **can** develop a market. One reason is that the existence of contractual commitments between the manufacturer and local parties helps to hold the parties to their commitment to humanitarian provision. Insisting that humanitarian aid cannot lead to gainful sales could therefore discourage manufacturers from offering such aid. - Indeed, emphasising the absence of a financial return could harm patients. A manufacturer who is not immediately persuaded to offer humanitarian aid might argue that most countries and markets offer at least **some promise** for financial gain and they intend to sell rather than give for free in those areas, and humanitarian provision is therefore not an option.   For these reasons, no principled distinction can be maintained between compassionate use and humanitarian provision. The two may differ in how long it takes to achieve commercial sales: under the EU compassionate use programme the road to commercial sales may naturally be shorter than in a country with a less developed programme and a younger general medical infrastructure. However, since the nuance here pertains only to timing, the arguments in favour of compassionate use provision explored in the main article apply equally to humanitarian aid | Prescribing a medicine for a condition other than what it was licensed/authorised for | A legal requirement before gaining marketing authorisation to ensure a drug’s safety and efficacy |
| **Disease** | A life-threatening or chronically or seriously debilitating condition | A life-threatening or chronically or seriously debilitating condition | Any indication for which the product is not licensed | Any |
| **License** | Therapy not yet licensed, or licensed but not yet available in the market | The **second distinction** often advanced between compassionate use and humanitarian aid is that compassionate use pertains to an **unlicensed** drug whereas humanitarian aid usually pertains to a **licensed** drug. However, the distinction is not watertight as compassionate use can concern both unlicensed drugs (before the marketing authorisation is granted) and licensed (after marketing authorisation is granted but before the drug is available in the market) | Licensed for other indication(s) | Drug under trial can be licensed for other indication(s) or not licensed |
| **Responsible party** | Prescribing physician with approval from regulatory authorities | Prescribing physician with approval from regulatory authorities | Prescribing physician | Trial sponsor with approval from the regulatory authorities |
| **Data collection** | In some Member States, some data are reported to the regulatory authorities | This is the **third distinction** often advanced between humanitarian aid and compassionate use. Data collection will be more established in areas of compassionate use, whereas countries benefitting from humanitarian aid may be less well-developed medical infrastructure for investigations and follow-up.  However, data collection under compassionate use is by no means automatic and depends on the engagement of the company and treating physician as well as specific national rules. Further, because a doctor or nurse is nearly always required for the provision of humanitarian aid – for example infusion-based treatments for lysosomal diseases – a manufacturer could request the collection of data at the time of administration. In any event, as a regulatory requirement and as part of pharmacovigilance, provision of humanitarian aid also mandates documentation of health events such as serious adverse events or unusual events.  These requirements represent a commitment that is much greater than the collection of specified data, such as biomarkers of disease activity or more routine determination of analytes present in blood.  The requirements also place an added burden on the manufacturer, who may feel that the local circumstances, clinical resources and engagement of personnel and patients themselves, would compromise their capacity to comply with regulatory procedures – and hence could jeopardise approval in that country or region | Spontaneous adverse events may be reported | Outcome measure and adverse event data are reported to the regulatory authorities |
| **Continued Access** | Regulation 726/2004 obligates providers to continue access once a compassionate use programme is started for an individual until the therapy is available in the market | Manufacturer determines length of provision but it is hoped to be available until the therapy is available in the market (and the patient can access it) | Access through prescription | Declaration of Helsinki stipulates that trial parties “should make provisions for post-trial access for all participants who still need an intervention identified as beneficial in the trial”. |
