# Supplementary material for: Compassionate use of orphan drugs
Source: Orphanet J Rare Dis. 2015 Aug 21;10:100. doi: 10.1186/s13023-015-0306-x (PMC4546220; doi:10.1186/s13023-015-0306-x)
Supplement: Additional file 2: Table S2. — Analysis of EU Regulation 726/2004. A table analysing the legal definition of compassionate use in the EU. (DOCX 80 kb) [file 13023_2015_306_MOESM2_ESM.docx]

**Additional file 2: Table S2. Analysis of EU Regulation 726/2004**

| **Issue** | **Description** |
| --- | --- |
| Background and objectives | Regulation 726/2004 provides a centralised (as opposed to a Member State led) procedure for authorising medicinal products.[21] This allows applicants to obtain a marketing authorisation from the EMA (assessed by the Committee for Medicinal Products for Human Use (CHMP)) that is valid throughout the EU and is a compulsory route for orphan medicinal products which are the focus of this article.[21] Article 83 of the Regulation provides an exemption from the marketing authorisation requirement on a compassionate use basis.  The objectives of Article 83 are to “meet, in particular, the legitimate expectations of patients (…) a common approach should be followed, whenever possible, regarding the criteria and conditions for the compassionate use of new medicinal products under MS’ legislation” (Recital 33 to the Regulation). The EMA has elaborated that the objectives of Article 83 are to:   - **Facilitate and improve the access** of patients in the European Union to compassionate use programmes; - **Favour a common approach** regarding the conditions of use, the conditions for distribution and the patients targeted for the compassionate use of unauthorised new medicinal products; and - **Increase transparency** between Member States in terms of treatment availability.[22] |
| What falls outside | The following fall outside the scope of the Regulation:   - Medicinal products which are not eligible for the centralised procedure; - Compassionate use on a named patient basis (as provided for in Article 5 of Directive 2001/83/EC); and - A medicinal product, which has already been authorised via the centralised procedure, even if the proposed conditions of use and target population are different from those of the marketing authorisation.[22] |
| Definition of compassionate use | Article 83(2) of the Regulation defines compassionate use as a Member State making a treatment available (i) for compassionate reasons (ii) to a group of patients (iii) with a chronically or seriously debilitating or life-threatening disease (such as cancer, HIV/AIDS, neurodegenerative disorders and auto-immune diseases[22]) (iv) who cannot be treated satisfactorily by an authorised medicinal product in the particular Member State[22], and, finally, (v) the therapy in question is the subject of an application for a marketing authorisation or undergoing clinical trials. |
| Interface with Member State law | **Elective aspects of the Regulation**  The Regulation is thought to provide only a loose legal framework. Recital 33 makes clear that the Regulation seeks a common approach “**under Member State legislation**”. The EMA too views Article 83 as complementary to legislation adopted on Member State level and as one which leaves Member States to implement and coordinate compassionate use programmes.[8,22] The EMA advises that Member States decide independently how and when to open such programmes according to national rules and legislation. Doctors who wish to obtain a promising medicine for one of their seriously ill patients will need to contact the relevant **national** authority in their respective country and follow the procedure that has been set up. The national authority keeps a register of the patients treated with the medicine within the compassionate use programme, and systems are in place to record any side effects reported by the patients or their doctors[8].  It is also thought that Member States can choose to seek an opinion from the CHMP regarding the conditions for compassionate use of a specific medicinal product which falls within the scope of Article 83(1) and 83(2)[22] although this is not expressly set out in Article 83. The CHMP simply makes optional recommendations to EU countries as to how to administer, distribute and use medicines for compassionate use, and to suggest which patients may benefit from compassionate use programmes.[8,22,23]  **Compulsory aspects of the Regulation**  What is not underlined in the EMA’s commentary is that the Regulation does nevertheless bind the Member States. An EU regulation is directly applicable law in Member States, meaning that it takes effect as law in Member States without the need for national implementing legislation.[24] As a result, where a Member State implements a compassionate use programme for an orphan medicial product or other therapy which is being approved via the central procedure, the Member State must follow Article 83. In practice, this means that:   - The Member State can only implement a compassionate use programme for a therapy that meets the criteria in Article 83(3) – see “Definition of compassionate use” above. It cannot for example implement a compassionate use programme where the condition is not chronically or seriously debilitating or life-threatening. As a result, all compassionate use programmes for orphan medicines (and other medicines for which authorisation is sought centrally from the EMA) exist to satisfy the same need on the same terms. - The Member State “shall” notify the EMA of the compassionate use programme (Article 83(3)) which has resulted in the data in Supplemental File 3. - The Member State “shall” also take account of any available opinions issued by the CHMP (Article 83(5)). |
| Role of the EMA/CHMP | As noted, the CHMP makes recommendations to EU countries as to how to administer, distribute and use medicines for compassionate use, and to suggest which patients may benefit from compassionate use programmes.[8,22,23]  The EMA notes that such recommendations aim to standardise compassionate use programmes across the EU and they may help make the conditions of existing compassionate use programmes clearer. However, the EMA notes that “such recommendations complement national legislation, and do not replace it. They also do not create any legal framework in the EU Member States. The recommendations are optional, and are only implemented by the Member States that wish to use them for their patients.” However, it should be noted that Article 83(5) of the Regulation obligates Member States to “take account of any available opinions” issued by the EMA.  The Regulation does **not** provide who can apply for an opinion by the CHMP. The EMA’s interpretation is nevertheless that only Member States, not manufacturers, can seek such an opinion. However, the EMA advises that “companies may, at their own initiative, inform the EMEA of compassionate use applications in MS(s), or of an ongoing procedure for compassionate use at national level. The EMEA may then contact the relevant MS(s) for information only”.[23] |
| Not a substitute for clinical trials | Compassionate use is not a substitute for clinical trials which the EU views as a source of essential information about the risk/benefit balance of a treatment. Patients should always be considered for inclusion in clinical trials before being offered compassionate use programmes.[22] |
| Continued access to treatment after marketing authorisation | Article 83(8) of the Regulation helpfully ensures that patients taking part in a compassionate use programme have access to the treatment between the time when a drug gains market authorisation and when it is placed on the market,[25] to avoid an abrupt pause in a potentially life-saving treatment. This type of bridging provision is known (in the clinical trial context) as an “expanded access programme”[8] and the same principle (to bridge two stages of provision) applies here. |
| Compassionate use need not be for free | The definition of compassionate use does not involve giving the drug away for free: for example the French national regime involves hospital pharmacies paying full price to manufacturers for the compassionate use treatments – indeed the companies set a 12% premium on compassionate use rates.[3] This however seems a limited experience, and ordinarily a compassionate use programme appears to involve donating a drug. For example Germany amended its compassionate use legislation in 2009 to demand that compassionate use drugs must be provided free of charge.[5] |
| Temporary measure | Article 83 makes no express reference to compassionate use being a temporary measure. Article 83(1) provides as follows: “*By way of exemption* from Article 6 of Directive 2001/83/EC Member States may make a medicinal product for human use belonging to the categories referred to in Article 3(1) and (2) of this Regulation available for compassionate use” (emphasis added).  However, Article 83(8) contemplates the treatment being available until launch (“Where a compassionate use programme has been set up, the applicant shall ensure that patients taking part also have access to the new medicinal product during the period between authorisation and placing on the market.”). This may imply that the EU compassionate use programmes are expected to end at this point. |
