# Supplementary material for: Compassionate use of orphan drugs
Source: Orphanet J Rare Dis. 2015 Aug 21;10:100. doi: 10.1186/s13023-015-0306-x (PMC4546220; doi:10.1186/s13023-015-0306-x)
Supplement: Additional file 3: Table S3. — Compassionate use programmes notified to EMA by Member States arranged by year. A table of compassionate use programmes notified to EMA by Member States, obtained from EMA via a freedom of information request. (DOCX 175 kb) [file 13023_2015_306_MOESM3_ESM.docx]

**Additional file 3: Table S3. Compassionate use programmes notified to EMA by Member States arranged by year. Yellow background indicates that the drug has orphan drug designation** [26] (Presentational format is the authors’ own)

| **Active substance** | **Therapeutic indication** | **2013** | **2012** | **2011** | **2010** | **2006-7** |  |
| --- | --- | --- | --- | --- | --- | --- | --- |
| Nintedanib (BIBF 1120) | Locally advanced or metastatic non-small cell lung cancer progressing after chemotherapy |  |  |  |  |  |  |
| Oseltamivir phosphate 100 mg | None indicated |  |  |  |  |  |  |
| Midostaurin (PKC412) | Aggressive Systemic Mastocytosis (ASM) |  |  |  |  |  |  |
| Regorafenib | (1) Gastrointestinal Stromal Tumors (GIST) after disease progression on or intolerance to imatinib and sunitinib (2) Patients with metastatic colorectal cancer previously treated with fluoropyrimidine-, oxaliplatin- and irinotecan-based chemotherapy |  |  |  |  |  |  |
| Sofosbuvir in combination with daclatasvir | Chronic Hepatitis C virus (HCV) infection in genotype 1 patients with failure on boceprevir or telaprevir based therapy |  |  |  |  |  |  |
| Sofosbuvir with Ribavirin and with or without Pegylated Interferon | (1) Chronic Hepatitis C virus (HCV) infection in pre or post liver transplantation  (2) Patients with Liver Transplantation and aggressive, recurrent Hepatitis C infection |  |  |  |  |  |  |
| Enzalutamide | Castration resistant metastatic prostate cancer in patients that have received Docetaxel therapy |  |  |  |  |  |  |
| Idebenone | Duchenne Muscular Dystrophy |  |  |  |  |  |  |
| TMC 207 (Bedaquiline) | Extensively Drug Resistant (XDR) or pre-XDR Mycobacterium tuberculosis (MTB) Pulmonary Infection |  |  |  |  |  |  |
| Teduglutide | Short Bowel Syndrome (SBS) |  |  |  |  |  |  |
| Zanamavir 10 mg/ml | Life-threatening influenza infection |  |  |  |  |  |  |
| [177]Lutetium‑DOTA[0]-Tyr[3]-Octreotate | Inoperable or metastasizing somatostatin-receptor-positive gastro-entero-pancreatic neuroendocrine tumours (GEP-NET) |  |  |  |  |  |  |
| Teriflunomide | Relapsing multiple sclerosis |  |  |  |  |  |  |
| Human Recombinant Tissue-Nonspecific Alkaline Phosphatase Fusion Protein (ENB-0040) | Very Severe Hypophosphatasia (HPP) |  |  |  |  |  |  |
| Human alpha-Mannosidase | Alpha-Mannosidosis |  |  |  |  |  |  |
| Pherubane | Dysphagia and urea cycle disorders |  |  |  |  |  |  |
| Dolutegravir | (HIV)-1 |  |  |  |  |  |  |
| Afatinib | Non-small-cell lung carcinoma (NSCLC) |  |  |  |  |  |  |
| Chlorhydrate de propranolol | Proliferating Infantile Hemangiomas |  |  |  |  |  |  |
| Decitabine | De novo or secondary acute myeloid leukaemia |  |  |  |  |  |  |
| Ruxolitinib | Symptomatic treatment of primary myelofibrosis |  |  |  |  |  |  |
| Crizotinib | Non-squamous carcinoma of the lung harbouring a translocation or inversion event involving the anaplastic lymphoma kinase (ALK) gene locus. |  |  |  |  |  |  |
| Lacosamid | Refractory epilepsy |  |  |  |  |  |  |
| Abirateron-Acetate | Metastatic advanced prostate cancer (castration resistant) |  |  |  |  |  |  |
| Fingolimod | Relapsing Remitting Multiple Sclerosis |  |  |  |  |  |  |
| Afamelanotid | Erythropoietic protoporphyria (EPP) |  |  |  |  |  |  |
| Cabazitaxel | Metastatic hormone refractory prostate cancer |  |  |  |  |  |  |
| Velaglucerase | Type I Gaucher disease |  |  |  |  |  |  |
| Thalidomide | Compassionate use indication not provided |  |  |  |  |  |  |
| Sorafenib tosylate | Metastatic RCC after failure to cytokine-based therapy |  |  |  |  |  |  |
| Sunitinib malate | Malignant gastrointestinal stromal tumours (GIST) and Metastatic renal cell carcinoma (MRCC) |  |  |  |  |  |  |
| Dasatinib | Chronic Myeloid Leukaemia (CML) and Acute Lymphoblastic Leukaemia (ALL) |  |  |  |  |  |  |
| Lenalidomide | Compassionate use indication not provided |  |  |  |  |  |  |
| Idursulfase | Compassionate use indication not provided |  |  |  |  |  | **Grand total** |
|  | **Total for the year** | 14 | 13 | 3 | 5 | 6 | 41 |
|  | **Total number of OMPs** | 6 | 4 | 0 | 3 | 4 | 17 |
|  | **Percentage of OMPs out of total** | 43% | 31% | 0% | 60% | 67% | 41% |
