# Supplementary material for: Compassionate use of orphan drugs
Source: Orphanet J Rare Dis. 2015 Aug 21;10:100. doi: 10.1186/s13023-015-0306-x (PMC4546220; doi:10.1186/s13023-015-0306-x)
Supplement: Additional file 4: Table S4. — Legal mechanisms for achieving compassionate use provision. A table elaborating on legal mechanisms for achieving compassionate use provision. (DOCX 74 kb) [file 13023_2015_306_MOESM4_ESM.docx]

**Additional file 4: Table S4. Legal mechanisms for achieving compassionate use provision**

| **Relationship between** | **Description** |
| --- | --- |
| The EU and company | There is presently no EU law that obliges an orphan company to provide drugs for free in the event of temporary problems preventing access.  One way to achieve this would be to amend Regulation 141/2000 which sets out the criteria for obtaining orphan designation. The amendment could state that orphan designation is maintained only if, once the company has obtained marketing authorisation, it donates the drug for free to those EU Members States that have a Gross Domestic Product below a very low level (for example Romania and Bulgaria) or are undergoing a severe recession (for example Greece where treatment cessation is feared to lead to death[12]) or are otherwise demonstrably unable to pay for the treatment.  This would be preferable over a criterion that a drug must be donated until it is accommodated within a Member State’s list of approved treatments, as that might encourage a Member State not to include orphan drugs in the formulary. The amendment would also appear to comply with EU law of which a central tenet is the free movement of goods: therapies would reach Member States presently unable to purchase them.  It may seem audacious to impose a legal obligation on a profit-making organisation to donate its product. But a precedent exists: UK energy companies are under a legal obligation to install free energy saving measures such as boilers and loft and cavity insulation to certain UK households; if they do not meet these targets, they are fined. Orphan legislation could provide that orphan companies can obtain tax relief for costs associated with compassionate use.  A downside is however that legislative amendment takes time and may meet with resistance from the industry, and would not assist patients in countries outside the EU. It may also drive up the price of drugs, as companies would factor the cost of compassionate use into the price of a drug it sets for the EU market, though this is likely already being done by manufacturers who presently (and laudably) donate drugs. In particular, wealthy EU countries may object that manufacturers would increase prices to the remaining EU countries in order to cover the cost of free supply, and in this way wealthier Member States would subsidise the healthcare budgets of less wealthy Member States. However, the amendment could in fact assist EU Member States by diminishing the appeal of health tourism (e.g. patient relocating to the UK to access an expensive rare disease treatment).  A further limitation is that the provision would only work within the EU and EU Member States would be slow to extend any such amendment into an obligation for EU Member States to provide free treatment *outside* the EU where free provision is often most desperately needed. |
| Two companies | An alternative is to create a legal obligation between two manufacturers to share the burden of compassionate provision. This appears difficult because no direct legal relationship exists between one company and another. However, the terms of engagement of a pharmaceutical interest group could specify that, where more than one company sells a drug for the same condition, each manufacturer should make its best effort to partake in a compassionate use program. This could be made part of the terms of participation in the interest group, such as the European Federation of Pharmaceutical Industries and Associations (EFPIA), and constitutions of national organisations such as the Association of the British Pharmaceutical Industry (ABPI).[27] The downside is that the wording would not cover a situation where only one drug exists for a condition, as it would not be in a company’s interest to impose a unilateral obligation on itself. |
| Patients and company | The third solution is to stipulate in national and international regulations governing clinical trials that patients have the right to a free supply following the trial, should governments or private health plans not cover it. But such amendment takes time. A quicker solution may be for patient organisations such as EURORDIS and NORD to demand the adoption of a standard contract between the company and each individual trial-participant. The standard wording could guarantee a free supply of the drug following the trial, even if the particular patient has been on a placebo, until the government or patient’s health plan covers it. As there are few sufferers of orphan conditions, most patients should be able to participate in such trials and therefore continue to access the drug. |
